# A three-dimensional ParF meshwork assembles through the nucleoid to mediate plasmid segregation

**DOI:** 10.1093/nar/gkw1302

**Published:** 2016-12-29

**Authors:** Brett N. McLeod, Gina E. Allison-Gamble, Madhuri T. Barge, Nam K. Tonthat, Maria A. Schumacher, Finbarr Hayes, Daniela Barillà

**Affiliations:** 1 Department of Biology, University of York, Wentworth Way, York YO10 5DD, UK; 2 Department of Biochemistry, Duke University Medical Center, Duke University, Durham, NC 27710, USA; 3 Faculty of Biology, Medicine and Health, The University of Manchester, Manchester M13 9PL, UK

## Abstract

Genome segregation is a fundamental step in the life cycle of every cell. Most bacteria rely on dedicated DNA partition proteins to actively segregate chromosomes and low copy-number plasmids. Here, by employing super resolution microscopy, we establish that the ParF DNA partition protein of the ParA family assembles into a three-dimensional meshwork that uses the nucleoid as a scaffold and periodically shuttles between its poles. Whereas ParF specifies the territory for plasmid trafficking, the ParG partner protein dictates the tempo of ParF assembly cycles and plasmid segregation events by stimulating ParF adenosine triphosphate hydrolysis. Mutants in which this ParG temporal regulation is ablated show partition deficient phenotypes as a result of either altered ParF structure or dynamics and indicate that ParF nucleoid localization and dynamic relocation, although necessary, are not sufficient *per se* to ensure plasmid segregation. We propose a Venus flytrap model that merges the concepts of ParA polymerization and gradient formation and speculate that a transient, dynamic network of intersecting polymers that branches into the nucleoid interior is a widespread mechanism to distribute sizeable cargos within prokaryotic cells.

## INTRODUCTION

The distribution of newly replicated genomes to daughter cells is a finely tuned process that requires high spatial precision and coordination with other cellular events. In eukaryotic cells, a large protein complex, the kinetochore, hitches sister chromatids to microtubules of the mitotic spindle that eventually drag them apart (1). Bacteria rely on a more parsimonious apparatus that consists of a NTPase, a DNA-binding protein and a *cis*-acting DNA site, known as a partition or centromere-like site. Genes encoding the two proteins together with the partition site constitute a segregation module. These modules are harbored by low copy number plasmids and chromosomes and fall into three categories according to the NTPase they encode, which can be either Walker-type, actin-like or tubulin-like (2).

Walker-type segregation modules are the most widespread and the only modules found also on chromosomes (3). The modules encode a Walker-type Adenosine triphosphatase (ATPase), ParA, and a site-specific DNA-binding protein, ParB, that contacts the centromere-like site and recruits ParA into a ternary nucleoprotein complex or segrosome. Within this class, two sub-types are recognizable: one characterized by large ParAs (∼250–400 residues) and canonical ParBs (∼320–360 residues) and the other including shorter ParAs (∼200 residues) accompanied by smaller DNA-binding proteins (≤100 residues) unrelated to ParB. ParA proteins exhibit weak ATPase activity that is stimulated by ParB and that is essential for DNA segregation (4–10). Some ParAs were shown to assemble into higher order structures *in vitro* (8,11–16) and form cooperative assembly frameworks *in vivo* (10, 11,16–20), whereas others do not cluster obviously into higher order oligomers (21–23). ParA proteins also display non-specific DNA binding activity, which allows them to associate with the nucleoid (9,10,16,22–30). The precise mechanism underpinning plasmid segregation mediated by ParA proteins remains elusive. Current evidence suggests two models, whose unifying theme is the use of the nucleoid as a scaffold for segrosome attachment. One model invokes the formation of a nucleoid-associated ParA filament whose growing tip stochastically captures a ParB–plasmid complex. Upon adenosine triphosphate (ATP) hydrolysis promoted by ParB, the ParA filament depolymerizes pulling the plasmid toward a pole of the nucleoid (20). The other model predicts a diffusion-ratchet mechanism in which ParA bridges the ParB–plasmid complex to the nucleoid and then a ParA gradient guides plasmid movement (23,27,29,30). Recently, a modified gradient model that takes into account the elastic force of the chromosome has been proposed for *Caulobacter crescentus* (28).

The multidrug resistance plasmid TP228 harbors a segregation module that includes the *parFG* genes and upstream partition site *parH* (3). ParF is a Walker-type ATPase of the ParA family and ParG is a ribbon-helix–helix DNA-binding protein (31,32) that contacts repeats in the centromere-like site (33). Purified ParF assembles into extensive filaments *in vitro* (8). ATP binding promotes higher order assemblies, whereas adenosine diphosphate (ADP) antagonizes it. Nucleotide association acts as a molecular switch turning on either assembly or disassembly of the protein. Crystal structures of ParF in different nucleotide-bound states suggest a mechanism that underlies polymer formation. ParF bound to ADP is monomeric, whereas ParF-ATP forms dimers, which assemble into dimer-of-dimers building blocks. These units then pack into linear polymers (34). Mutations that disrupt the ParF interfaces in the polymer ablate plasmid segregation (34). ParG displays an N-terminal flexible tail that carries out two separate functions: it stimulates ParF ATP hydrolysis via an arginine finger-like motif and promotes the formation of ParF polymer bundles *in vitro* (35).

Here we show that ParF dynamically relocates between the poles of the nucleoid in *Escherichia coli*, thereby transporting the ParG–plasmid complex. This process is driven by cycles of ATP binding and hydrolysis. Perturbations of the ATP conversion cycle, caused by amino acid changes in either ParF or ParG, disrupt ParF location and dynamics. Although necessary, oscillation is not sufficient to effect plasmid segregation, as shown by a hyperoscillating ParF mutant, whose ATPase activity is no longer responsive to ParG. This observation is a departure from the canons established by previous findings on pB171 ParA (17,20) and ParA of plasmid F (36).

Strikingly, super resolution three-dimensional (3D) structured illumination microscopy shows that ParF assembles into a 3D meshwork that permeates the nucleoid and traps ParG–plasmid complexes within the chromosome region. Interestingly, in a mutant background in which the ParG arginine finger-like activity is compromised, the wild-type ParF protein no longer cooperatively self-assembles into the meshwork structure, but instead accumulates on the ParG–plasmid complexes. The results indicate that the entire nucleoid space is surveyed by the ParF partition protein and that compromised ATP kinetics undermine ParF structure and function. In view of these findings, a Venus flytrap novel mechanism for plasmid capture and segregation mediated by ParA proteins is proposed.

## MATERIALS AND METHODS

### Plasmids

The plasmids used for partition assays and microscopy (pBM20 series) are based on the pFH450/pFH554 plasmids (3) and harbored the TP228 segregation module (∼1.2 kb) with either wild-type or mutant *parF* or *parG* genes in which the *parG* gene was fused to the gene encoding the mCherry protein (Clontech). The copy number of the plasmid is estimated to approximately five per cell based on published information (21). The plasmid pBM22 contained the *parFG-mCherry-parH* module and a *lacO*_120_ array (37). A plasmid encoding the ParF-Emerald fusion was constructed in two steps: first, *parF* was cloned in frame with the gene encoding Emerald in plasmid pPT100 (38). Then the *parF-emerald* fusion gene was amplified by polymerase chain reaction and cloned into pBAD30 under the control of an arabinose-inducible promoter. Alleles containing point mutations in *parF* were constructed by swapping the fragment carrying the wild-type sequence with the fragments containing the mutations. The plasmid pBAD-LacI-EBFP2 was constructed by cloning a truncated version of *lacI* that is missing the last 12 codons in frame with the gene encoding EBFP2 in pBAD-ebfp2 (39). The plasmid expressing *parG-mCherry* under the control of P_tac_ was constructed by cloning the l*acI^q^*-Ptac promoter and the *parG-mCherry* fusion gene into pCDFDuet-1 vector (Novagen).

### Microscopy

*Escherichia coli* BW25113 transformants were grown in 1 ml of M9 glucose medium supplemented with antibiotic(s) for 1 h at 37°C. As the selective pressure was maintained, these experiments were performed to investigate plasmid localization rather than to observe plasmid loss. When LacI-EBFP2 was imaged, *E. coli* JW0336 was used. Cultures were induced with L-arabinose at a final concentration of 0.02% and grown for 2–3 h at 30°C. Cell pellets were resuspended in 50 μl of M9 glucose with 0.02% arabinose without antibiotics. Less than 0.5 μl were placed on 1.2% agarose M9 glucose pads (with 0.02% arabinose and/or 2 μg ml^−1^ DAPI where indicated) and sealed using a geneframe (ABgene) and a coverslip. Confocal microscopy was performed using a Zeiss LSM710 or LSM780 microscope. Wide-field fluorescence microscopy was performed with an Olympus IX70 Inverted System Microscope and a Photometrics CoolSNAP HQ CCD. 3D-SIM super resolution microscopy employs patterned illumination to excite the sample. The resulting emission is the product of the structured illumination pattern superimposed on that of the sample. This brings into the resolvable range high-resolution information that would otherwise be beyond the diffraction limit. Structured illumination microscopy was acquired on the DeltaVision OMX imaging system V2.2 (Applied Precision Inc.) with four solid-state multimode lasers (405, 488, 593 and 635 nm). The OMX V2.2 had an Olympus UPlanSApo 100_B_ 1.4 NA oil objective. Samples were sectioned using a 125 nm z-step and images reconstructed using Volocity software package (Perkin Elmer).

### Plasmid partition assays

Partition assays were performed as described elsewhere (3) using the same medium (M9 glucose) and conditions (±0.02% arabinose) adopted for microscopy. The relevant plasmid-bearing strains were grown for ∼25 generations without chloramphenicol selective pressure. Plasmid retention was then determined by replica plating colonies to agar medium in the presence and absence of antibiotic. The values presented are the means of at least three independent tests.

### Fluorescence polarization

Experiments were performed with a PanVera Beacon 2000 fluorimeter at 25°C using 5΄ fluoresceinated double stranded oligonucleotides at a final concentration of 1 nM and increasing concentrations of ParF. The oligonucleotides used for ParF binding were: 20-mer (5΄ – AATTACTCAATTACTCAATT - 3΄), 42-mer (5΄-CAAGAAATAAACCAAAAATCGTAATCGAAAGATAAAAATCTG – 3΄) and 13-mer (5΄ - CAAGAAATAAACC – 3΄). The studies were conducted in a buffer containing 150 mM potassium glutamate, pH 7.5, 5 mM magnesium acetate and 5 mM nucleotide (ADP or ATP). The salt-dependency studies were conducted with the 20-mer oligonucleotide in the same buffer, but the potassium glutamate concentration ranged from 50 to 350 mM. Samples were excited at 490 nm and fluorescence emission was measured at 520 nm. The data were analyzed with KaleidaGraph and fitted to a simple bimolecular binding model by nonlinear regression.

### Western blot

*Escherichia coli* BW25113 transformants harboring the double plasmid system were grown in M9 minimal medium at 37°C for 1 h. The culture was induced with 0.02% L-arabinose and grown for 3 h at 30°C. Cells were centrifuged at 5000 rpm at 4°C for 10 min, resuspended in 1 ml of binding buffer (20 mM Tris-HCl, pH 7.5, 500 nM NaCl, 15 mM imidazole, 10% glycerol) and sonicated. The extract was centrifuged at 13 000 rpm, 4°C for 30 min. A total of 60 μl samples of the supernatant together with aliquots of purified ParF and ParG were loaded onto a 12% sodium dodecyl sulphate polyacrylamide gel. The gel was subjected to immunoblotting and the proteins were detected by using affinity-purified anti-ParF and anti-ParG antibodies (31).

## RESULTS

### Tracking the *trans*-acting factors of the TP228 segrosome in live *E. coli* cells

Subcellular localization of ParF and ParG in *E. coli* was investigated using a two-plasmid system. One plasmid was a segregation probe vector harboring the *parFGH* module, in which *parG* was fused to the gene encoding mCherry fluorescent protein and expressed from its native promoter. A second plasmid carried *parF* fused to the gene encoding the monomeric green fluorescent variant Emerald under control of the arabinose-inducible promoter P_BAD_. Plasmid partition assays, performed in the same conditions used for microscopy, established that both fusion proteins were functional: plasmids carrying native *parG* or the *parG-mCherry* allele were equally stable (∼65% retention after ∼25 generations of non-selective growth). ParF-Emerald activity was tested in multiple partition assays. First, when *parF-emerald* was expressed at the level used in fluorescence microscopy, it partially complemented a *parF* deletion raising the retention of the plasmid from 0 to 32%. Second, the fusion improved the partition of an otherwise unstable plasmid harboring a module encoding the ParF-K15Q mutant (8) whose retention increased from 1 to 23%. Importantly, in the two-plasmid system, *parF-emerald* expression does not affect the plasmid carrying the *parFG-mCherry-parH* module, whose stability is ∼65% both in the presence and absence of the plasmid harboring *parF-emerald*. Western blots on cells harboring the two-plasmid system showed that the fluorescent fusion proteins were full-length and that the level of ParF-Emerald was very similar to that of ParF (Supplementary Figure S1).

### ParG coalesces into foci that colocalize with *parFGH* plasmids

To determine the subcellular position of ParG, cells expressing the *parFG-mCherry-parH* locus were imaged by fluorescence microscopy and ParG-mCherry foci were observed (Supplementary Figure S2A). To investigate where these foci localized in relation to the plasmids, an array of *lac* operator sites, *lacO*_120_, was inserted into the plasmid carrying the *parFG-mCherry-parH* module. The resulting construct was transformed into cells containing a second plasmid expressing *lacI-ebfp2* encoding the Lac repressor fused to an enhanced version of Blue Fluorescent Protein 2 from the P_BAD_ promoter. LacI-EBFP2 bound the *lacO* sites, forming compact blue foci (Supplementary Figure S2A) that mark the position of plasmids harboring the segregation module. ParG-mCherry foci overlapped with the LacI-EBFP2 blue foci (Supplementary Figure S2A), indicating that the ParG-mCherry signal represents the protein bound to the *parH* site on the plasmids. In control experiments performed with a *parFGH* plasmid lacking the *lacO* array, no blue foci were observed and LacI-EBFP2 instead bound diffusely to the nucleoid (Supplementary Figure S2B). Further evidence supporting the colocalization of ParG with plasmid foci was provided by a complementation experiment. Cells that harboured two plasmids were imaged: one contained a disrupted partition module with a frameshift mutation in *parG*, and another expressed *parG-mCherry* from the P_tac_ promoter. ParG-mCherry foci were visible in the presence but not in the absence of the plasmid with the partition module (Supplementary Figure S2C and D). In the latter scenario, ParG-mCherry was distributed throughout the nucleoid. Overall these results establish that ParG colocalizes with *parFGH* plasmids, which reflects ParG binding to the *parH* site. Thus, ParG-mCherry foci were used as ‘trackers’ of *parFGH* plasmids to investigate their position in cells hosting wild-type or partition-defective plasmids.

### ParF defines plasmid positioning in the cell

**Figure 1.**
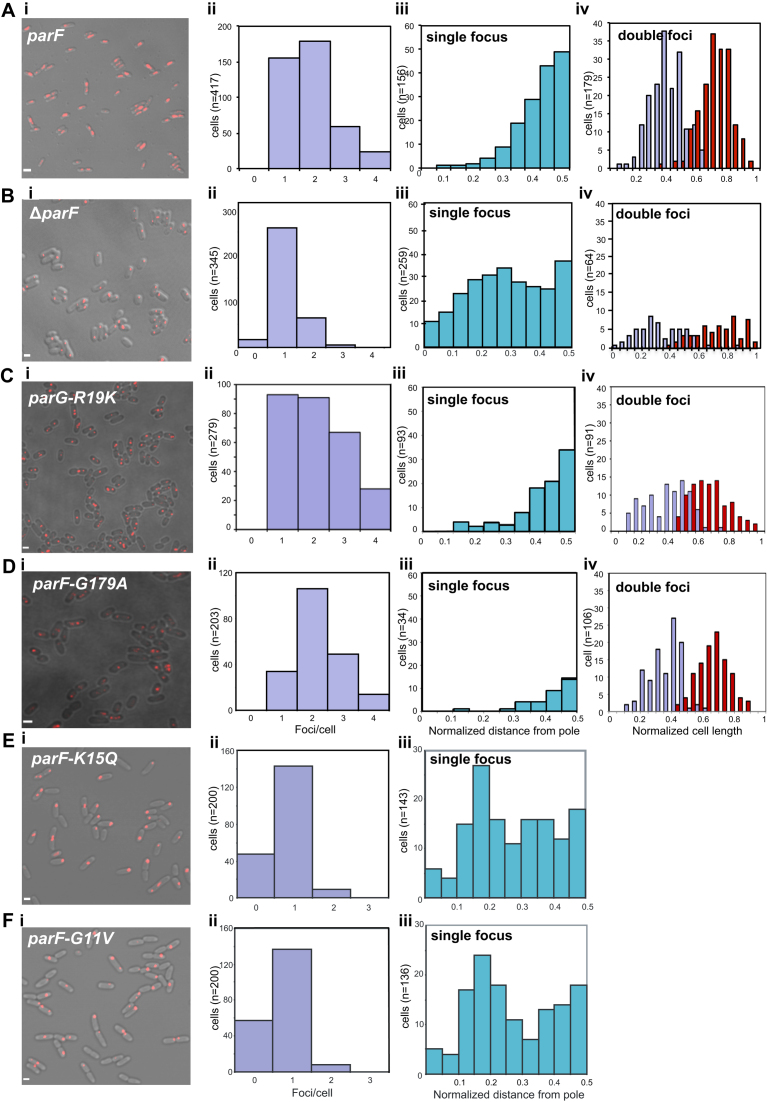
ParF-mediated positioning of *parFGH* plasmid. (**A–F**) Imaging and statistical analysis of ParG foci in *Escherichia coli* cells transformed with a plasmid expressing the indicated *parF* or *parG* allele from the *parFG-mCherry-parH* module and a plasmid expressing the same *parF-emerald* allele from the P_BAD_ arabinose inducible promoter, or empty pBAD30 vector in the case of Δ*parF*. (i) Representative field of view, scale bar = 2 μm in A, = 1 μm in B–F. (ii) Distribution of ParG foci per cell in a population of individual cells (*n*) measured from collapsed Z-stack images. (iii) Position of ParG foci along the long axis of cells with a single focus from the population shown in panels A–F ii. Positions are normalized relative to the closest cell pole. (iv) Position of ParG foci along the long axis of cells displaying two foci from the population shown in panels A–D ii. Cells were grown in M9 glucose supplemented with 0.02% L-arabinose in the presence of antibiotics.

Cells harboring the plasmid with the wild-type *parFG-mCherry-parH* module mainly exhibited 1–3 plasmid foci (Figure 1A, i and ii). In cells with one focus, the ParG-mCherry spot was positioned most frequently near mid-cell (Figure 1A, iii). When two plasmid foci were present, they were situated at one quarter and three quarter positions on the long axis of the cell (Figure 1A, iv). In contrast, cells with a segregation-impaired plasmid due to a *parF* truncation (Δ*parF*) displayed a single, randomly located plasmid focus (Figure 1B, i–iv). Time-lapse microscopy experiments showed that the plasmid focus was excluded from the nucleoid and visible at the tip of the nucleoid or between two nucleoids (Supplementary Movies S1 and 2). Alleles encoding ParF with a change in conserved residues of the Walker A motif, G11V and K15Q, abolish plasmid partition (8). Plasmid foci number and position in cells grown under selective pressure and carrying these mutant genes showed patterns very similar to that of cells harboring the Δ*parF* plasmid (Figure 1E and F). Notably, most of the cells exhibited a single focus, suggesting that post-replication plasmid separation does not occur. Furthermore, the single focus observed in mutant backgrounds appeared sharper and consistently more compact than foci seen in cells containing the wild-type partition locus (Supplementary Figure S3B and C). These results indicate that both plasmid segregation and positioning are disrupted in the absence of a functional ParF. Thus ParF is necessary to achieve correct localization of the plasmid prior to cell division.

### ParF dynamically relocates within the nucleoid

**Figure 2.**
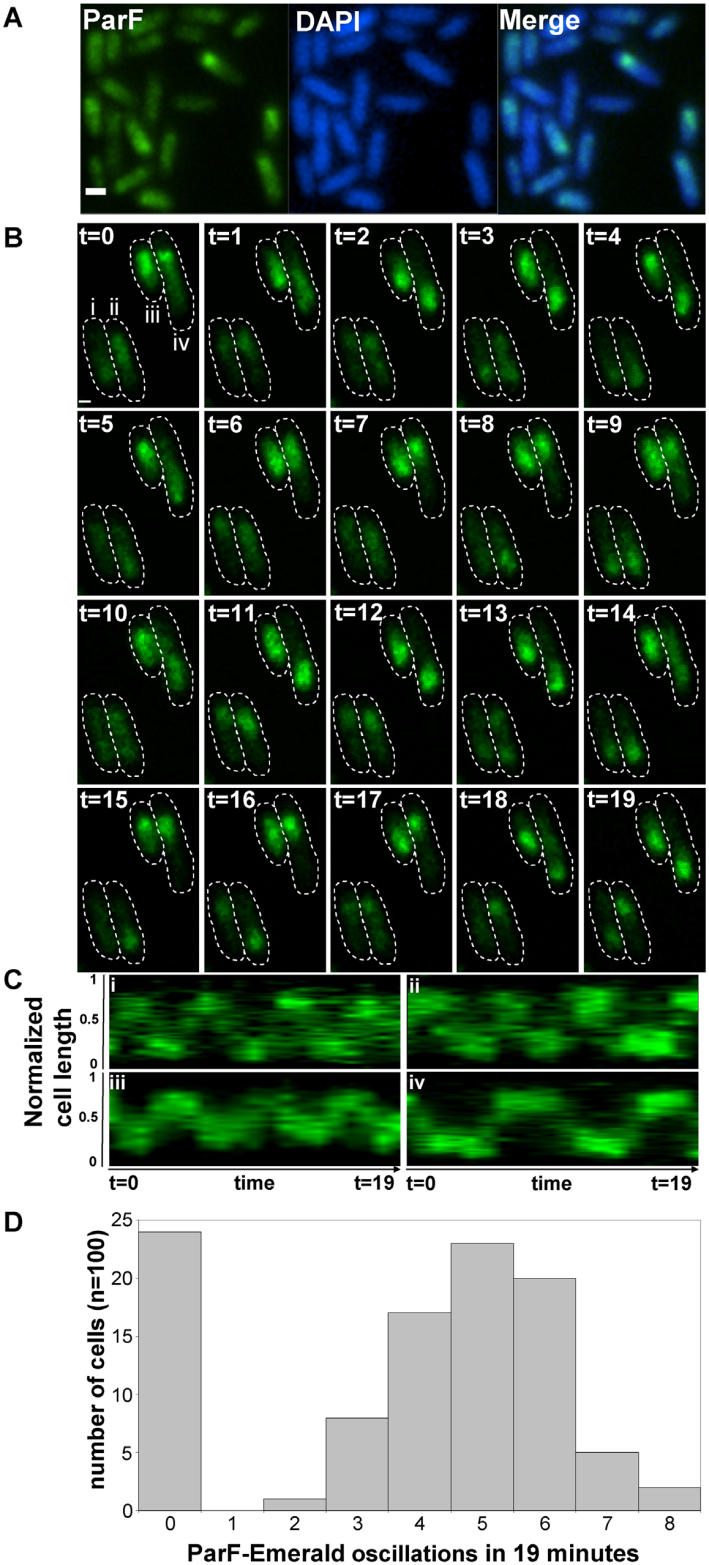
ParF coalesces into a cloud-like structure oscillating between the poles of the nucleoid. (**A**) Fluorescence microscopy snapshot of *Escherichia coli* cells transformed with the plasmid containing the *parFG-mCherry-parH* module and a plasmid expressing *parF-emerald* from the P_BAD_ promoter. ParF-Emerald (left), DAPI-stained nucleoid (middle) and merge image (right), scale bar = 0.5 μm. (**B**) Time-lapse fluorescence microscopy images of the same *E. coli* strain described in panel A showing the dynamic relocation of ParF-Emerald. Cell boundaries (dashed lines) were overlaid from bright field images. Time in minutes, scale bar = 0.5 μm. (**C**) Kymograph showing the movement of the ParF-Emerald signal along the cell length over time for the four cells (i, ii, iii and iv) shown in panel B. (**D**) Histogram illustrating the frequency of ParF-Emerald oscillations determined by constructing kymographs for 100 individual cells of the strain in panel B.

Next, the subcellular localization of ParF-Emerald was examined in populations expressing *parF-emerald* in the presence of the plasmid harboring the *parFG-mCherry-parH* module. The ParF signal appeared as an extended structure overlapping one pole of the nucleoid (Figure 2A). The ParF patches exhibited a compact head followed by a more diffuse tail. The head occupied the extreme edge of the nucleoid. In the absence of the plasmid with the partition locus, ParF-Emerald coated the chromosome uniformly, thus retaining its nucleoid localization, but not its asymmetric distribution (Supplementary Figure S3A). In contrast to wild-type, the signal of Walker A mutants ParF-G11V and ParF-K15Q was spread evenly throughout the cell, both in the absence and presence of the *parFGH* locus expressing the same mutant allele (Supplementary Figure S3A–C). Interestingly, the ParG-mCherry signal superimposed on that of wild-type ParF-Emerald, whereas it formed sharp foci silhouetted over the diffuse signal of ParF-G11V and ParF-K15Q (Supplementary Figure S3B and C). As ParF self-associates into higher order structures *in vitro* (8,34,35), the hazy ParF-Emerald signal may represent dynamic assemblies in transit over the nucleoid. To test this hypothesis we examined the signal using time-lapse microscopy. ParF patches oscillated between nucleoid poles in a majority of cells (Figure 2B and Supplementary Movie S3). Initially the signal appeared compact and round at one pole. Then it began to stretch laterally toward the nucleoid center, migrating soon after across the chromosome and reaching the opposite end, where the signal appeared again as a tight structure. Kymographs showing the position of ParF along the nucleoid length over time in one hundred cells revealed that a journey from pole to pole typically occurred every ∼4–6 min in most cells (Figure 2C and D). No dynamic relocation of ParF was observed in cells expressing *parF-emerald* in the absence of the TP228 partition locus (Supplementary Figure S4A). ParF-G11V and ParF-K15Q did not exhibit redistribution of their diffuse signal over time (data not shown). As these mutants are defective in ATP binding and hydrolysis resulting in disrupted polymerization dynamics (8), the data indicate that ATP association determines nucleoid localization of ParF and that impairment of correct assembly-disassembly dynamics abolishes ParF oscillation.

### Synchronous tracking of ParG in the wake of ParF

**Figure 3.**
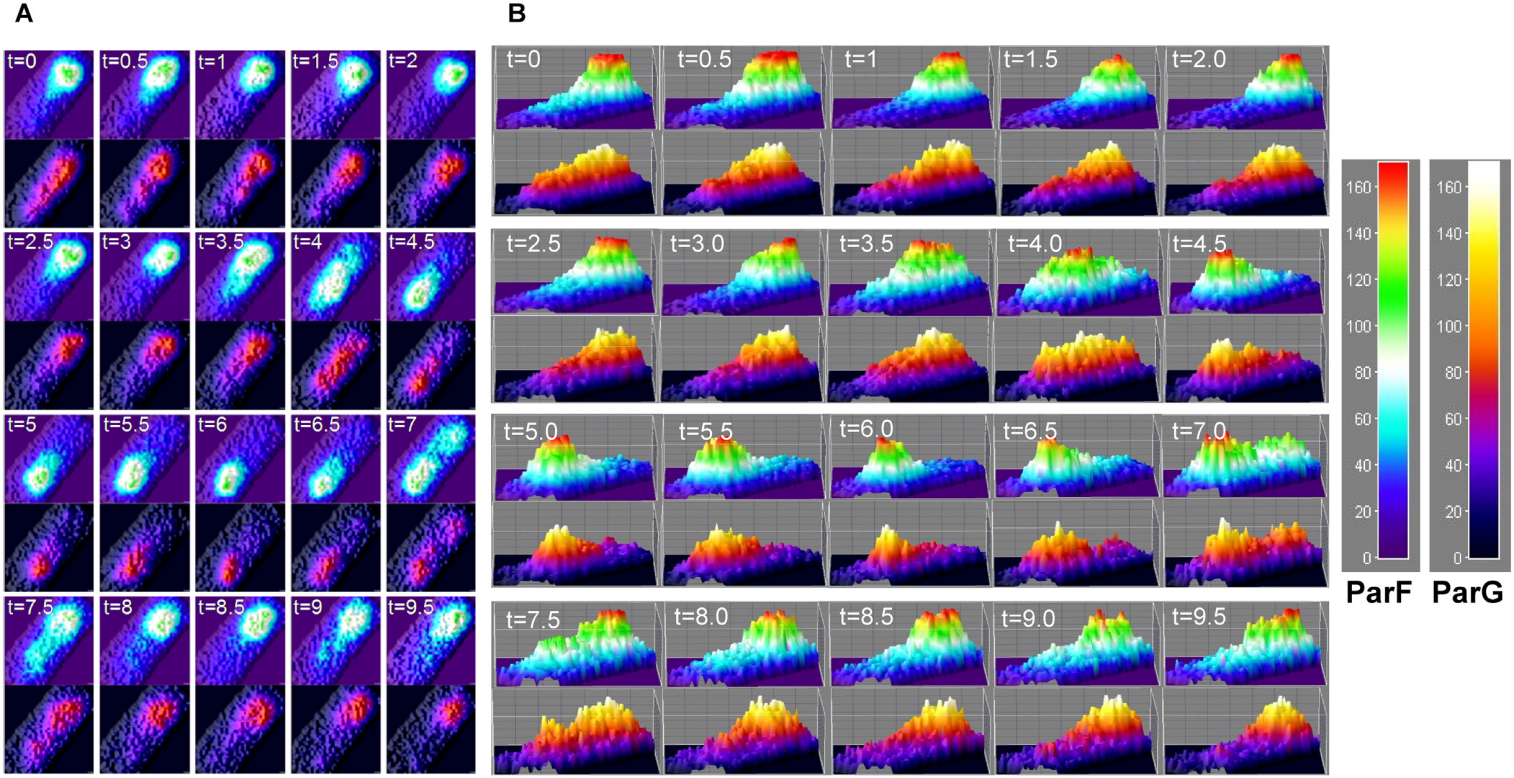
Movement of ParG-mCherry in relation to ParF-Emerald oscillation. Pixel intensity contour plots for the ParF and ParG signals from time-lapse microscopy images of a representative *Escherichia coli* cell harboring the segregation probe vector carrying *parFG-mCherry-parH* and the plasmid expressing *parF-emerald* (**A**) and quantitation of the relative fluorescent signals for ParF-Emerald and ParG-mCherry (**B**). One hundred cells were analy*z*ed and all showed synchronous oscillation of ParF and ParG with the exception of 17 cells that did not show ParF relocation. Time in minutes.

The localization of ParG in relation to ParF was investigated. In cells expressing *parF-emerald*, ParG-mCherry appeared as patches analogous to and overlapping those observed for ParF (Supplementary Figure S3B). These snapshot images suggest that ParG-plasmid complexes transit with ParF across the nucleoid. To better visualize the movement of ParG relative to ParF oscillations, pixel intensities of both ParF-Emerald and ParG-mCherry from time-lapse experiments were rendered into contour plots of individual nucleoids (Figure 3A and B). For clarity the two channels are shown separately. As described, the ParF signal oscillated across the nucleoid. The images and relative signal quantitations reveal that ParG and the associated plasmid move in synchrony with or lag shortly behind ParF. Cells at time zero exhibited a compact ParF-Emerald signal at one pole of the nucleoid, while the ParG-mCherry signal was comet-shaped with the tail of the comet extending beyond the contour of the ParF-Emerald signal (Figure 3A). However, whereas the ParF-Emerald signal remained essentially unchanged in the following four time frames, the ParG-mCherry signal contracted to a round focus overlapping the ParF signal. The discrete ParF-Emerald structure then reorganized stretching toward the nucleoid center before migrating to the opposite nucleoid pole. The ParG fluorescence synchronously tracked ParF with the two proteins showing superimposing intensity maxima (Figure 3B). Once at the new pole, the overlying signals remained still for a few time frames, before beginning the next race to the opposite nucleoid edge. Overall, a pattern of fully synchronized oscillation of the ParF and plasmid-bound ParG signals was apparent. The results indicate that ParG-plasmid complexes move in concert with the ParF patches within the nucleoid boundary.

### ParF relocation across the nucleoid is dependent on ParG and partition site *parH*

To gain insights into the role played by ParG in ParF oscillation, a plasmid carrying a non-functional partition module was used. This plasmid is segregationally unstable exhibiting a retention rate of ≤2% in the absence of selective pressure due to truncation of the C-terminus of ParG (3). The ParF-Emerald signal was static and evenly distributed over the nucleoid in cells harboring this plasmid together with the second plasmid expressing *parF-emerald* (Supplementary Figure S4B, *top row*). This result demonstrates that ParG is a key player in effecting ParF oscillation. To investigate whether providing ParG *in trans* would reconstitute oscillation, a third plasmid expressing *parG-mCherry* from the P_tac_ promoter was introduced: uneven localization and oscillation of ParF-Emerald across the nucleoid were restored (Supplementary Figure S4B, *middle row* and Supplementary Movie S4). In cells expressing *parF-emerald* and *parG-mCherry* in the absence of the plasmid carrying the partition site *parH*, ParF-Emerald homogeneously coated the nucleoid and did not oscillate (Supplemenatary Figure S4B, *bottom row*). Thus, ParF dynamic behavior is dependent on both ParG and the *parH* site.

### ParG stimulation of ParF ATPase activity mediates ParF oscillation

**Figure 4.**
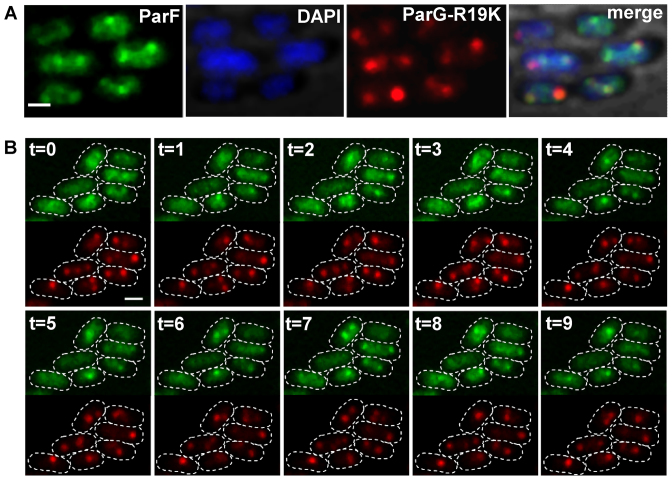
ParF oscillation is dependent on stimulation of ATP hydrolysis by ParG. (**A**) Fluorescence microscopy snapshot of *Escherichia coli* cells carrying a plasmid with *parG-R19K* mutant allele in the partition module and a plasmid expressing *parF-emerald*. Individual microscopy channels and a merge image with bright field are shown. Scale bar = 1 μm. (**B**) Time-lapse images of cells of the same *E. coli* strain as described in panel A. ParF-Emerald and ParG-R19K-mCherry channels (top and bottom, respectively) were imaged at 1 min intervals. Cell boundaries (dashed lines) were overlaid from the bright field images. Scale bar = 0.5 μm.

The ParG N-terminus harbors an arginine, R19, that is part of an arginine finger-like motif that stimulates ParF ATP hydrolysis. A ParG-R19K mutant is impaired in stimulation of ATPase activity and results in decreased plasmid stability indicating that this ParG-mediated function is a key regulatory process for segregation (35). Cells carrying plasmids with the *parG-R19K* allele in the *parFGH* locus and expressing *parF-emerald in trans* were observed under the presence of selective pressure. Surprisingly, the ParF-Emerald signal did not assemble into the usual extended structure onto the nucleoid, but distinctively most of the protein coalesced into foci that were not observed in any other mutant background (Figure 4). The ParF signal did not oscillate despite being wild-type (Figure 4A and B). The ParF foci colocalized with the ParG–plasmid complexes and showed some mobility accompanied by reorganization of the fluorescent signal over time. Most cells displayed one to three ParG-plasmid foci (Figure 1C, i and ii). Single ParG-R19K foci localized at midcell as observed in the wild-type background (Figure 1C, iii). In contrast, in cells containing two foci, they were found at all locations along the nucleoid with no bias toward one and three quarter positions. These data indicate that ParF oscillation is dependent on stimulation of its ATPase activity by the ParG–plasmid complex. Lack of stimulation causes accumulation and locking of ParF on ParG–plasmid complexes. In the absence of ParF relocation, plasmid positioning is disrupted.

### A hyperactive ATPase ParF mutant displays an increased frequency of cross-nucleoid oscillations

**Figure 5.**
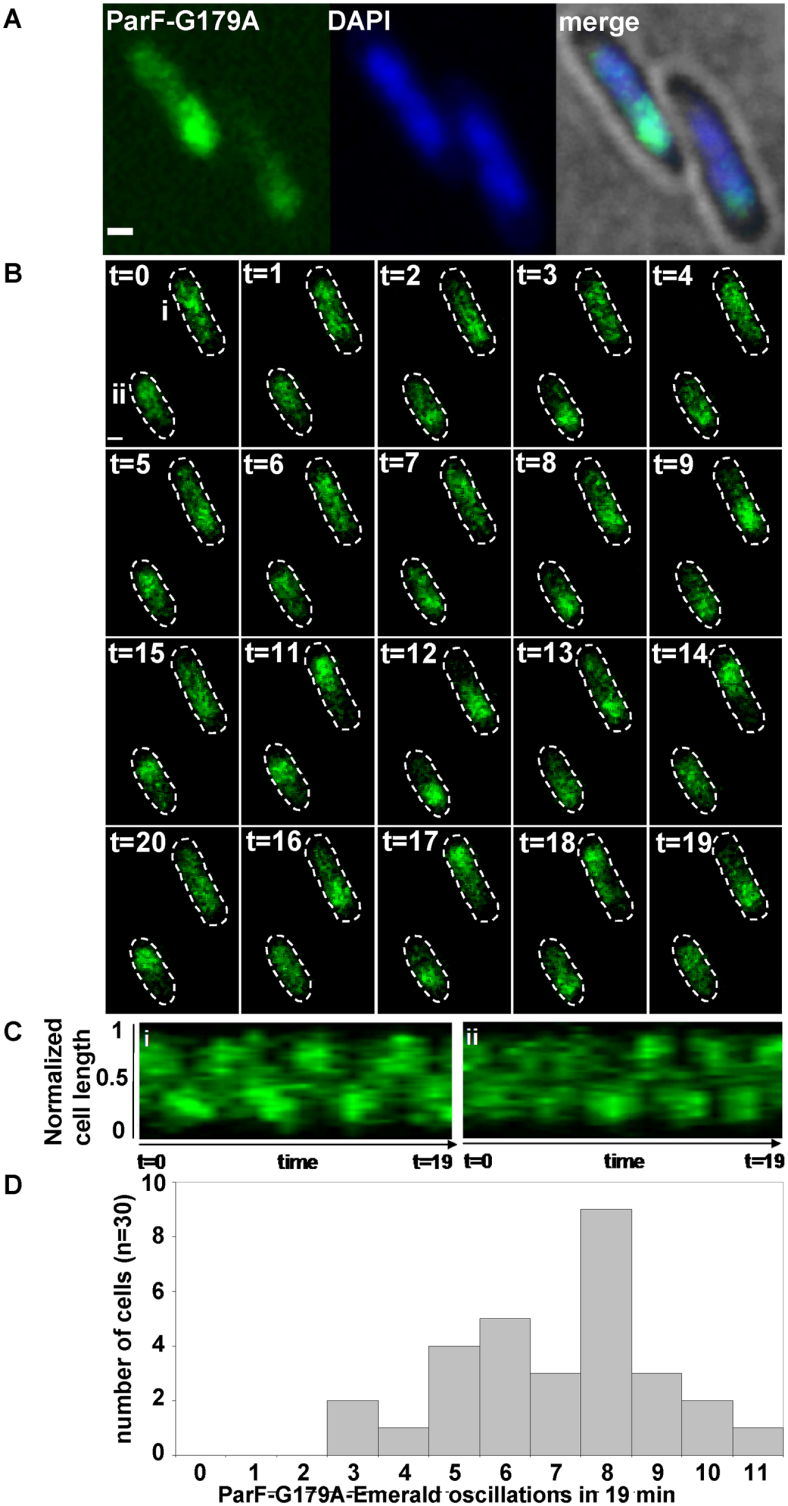
Hyperactive ATP hydrolysis results in increased cross-nucleoid oscillation frequency of the ParF-G179A mutant. (**A**) Fluorescence microscopy snapshot of *Escherichia coli* cells carrying a plasmid with *parF-G179A* mutant allele in the partition module and a plasmid expressing *parF-G179A-emerald*. ParF-G179A-Emerald, DAPI-stained nucleoid and merge image with bright field are shown. Scale bar = 0.5 μm. (**B**) Time-lapse images of the same *E. coli* strain described in panel A. Cell boundaries (dashed lines) were overlaid from bright field images. Time in minutes, scale bar = 0.5 μm. (**C**) Kymograph showing the movement of ParF-G179A-Emerald along the cell long axis over time in cells (i and ii) labeled at *t* = 0 in panel B. (**D**) Populations of cells showing the indicated number of ParF-G179A-Emerald pole-to-pole oscillations over 19 min determined from kymographs of ParF-G179A-Emerald transits in cells of the strain described in A.

As ParF ATPase activity is pivotal in mediating both ParF relocation and plasmid segregation, we investigated localization and dynamics of ParF-G179A, a mutant showing hyperactive ATP hydrolysis and defects in plasmid partitioning (40). In terms of kinetic properties, ParF-G179A ATPase activity has a *k*_cat_ = 14, which is almost 10-fold higher than that of wild-type ParF (*k*_cat_ = 1.6) and the activity is not stimulated by ParG (40). The *parF-G179A-emerald* allele was expressed in the absence and presence of the plasmid carrying the *parFG-mCherryH* locus with the same mutation in *parF*. In both instances, ParF-G179A localization was identical to that of wild-type ParF: in the absence of the partition module, ParF-G179A homogeneously coated the nucleoid (Supplementary Figure S3A), whereas it associated with one pole of the nucleoid in the presence of the plasmid harboring the segregation components (Figure 5A). Time-lapse experiments revealed that ParF-G179A oscillates between nucleoid poles like wild-type ParF (Figure 5B and Supplementary Movie S5). Remarkably, the hyperactive ATP hydrolysis induces a higher oscillation frequency compared to wild-type ParF with the majority of signals exhibiting pole-to-pole journey times of 2–3 min compared to 4–6 min for wild-type ParF (Figure 5C and D). In addition, ParF-G179A oscillation occurred in every cell in a field of view, whereas ParF did not oscillate in ∼25% of cells. In cultures grown in the presence of selective pressure, most cells contained two ParG-plasmid foci (Figure 1D, i and ii) that were more compact than those observed with a wild-type partitioning module. Surprisingly, the positions of single and double ParG foci relative to cell length did not differ between ParF-G179A and wild-type ParF (Figure 1D, iii and iv). It seems that a wild-type-like plasmid positioning is achieved by ParF-G179A, but the timing of partitioning events is disrupted. This mutant phenotype shows that responsiveness to ParG stimulation and finely tuned ATPase kinetics are key to effect plasmid segregation and that ParF nucleoid localization as well as oscillation are not sufficient *per se* to warrant plasmid partitioning.

### A three-dimensional ParF meshwork permeates the nucleoid to mediate plasmid segregation

**Figure 6.**
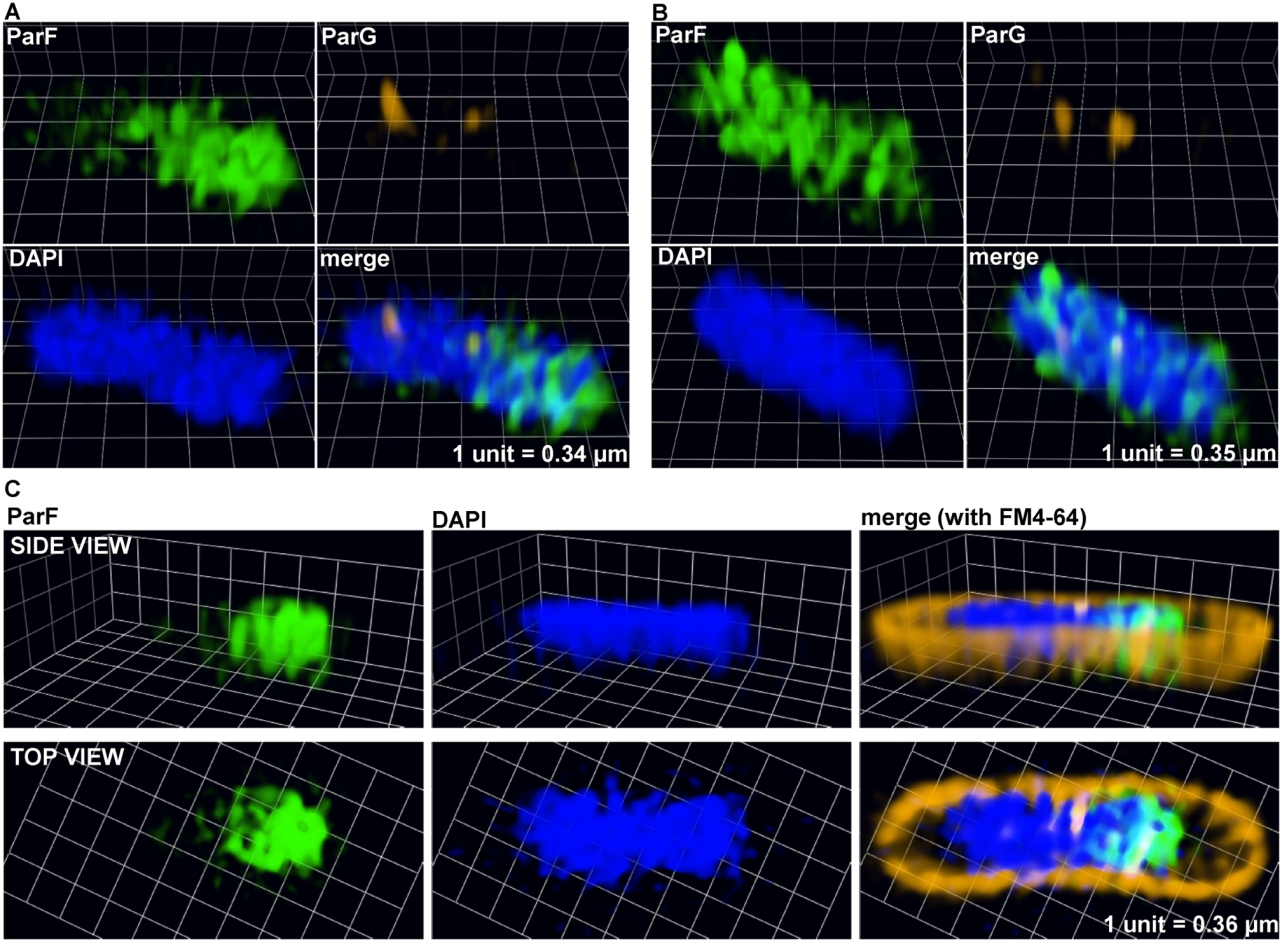
A three-dimensional (3D) ParF meshwork permeates the nucleoid. (**A–C**) Three-dimensional rendering of 3D-SIM images of live *Escherichia coli* cells harboring the plasmid containing the *parFG-mCherry-parH* module and the plasmid expressing *parF-emerald*. The cross section of a cell interior with side and top views is shown in panel C. Individual channels and overlay images are shown.

**Figure 7.**
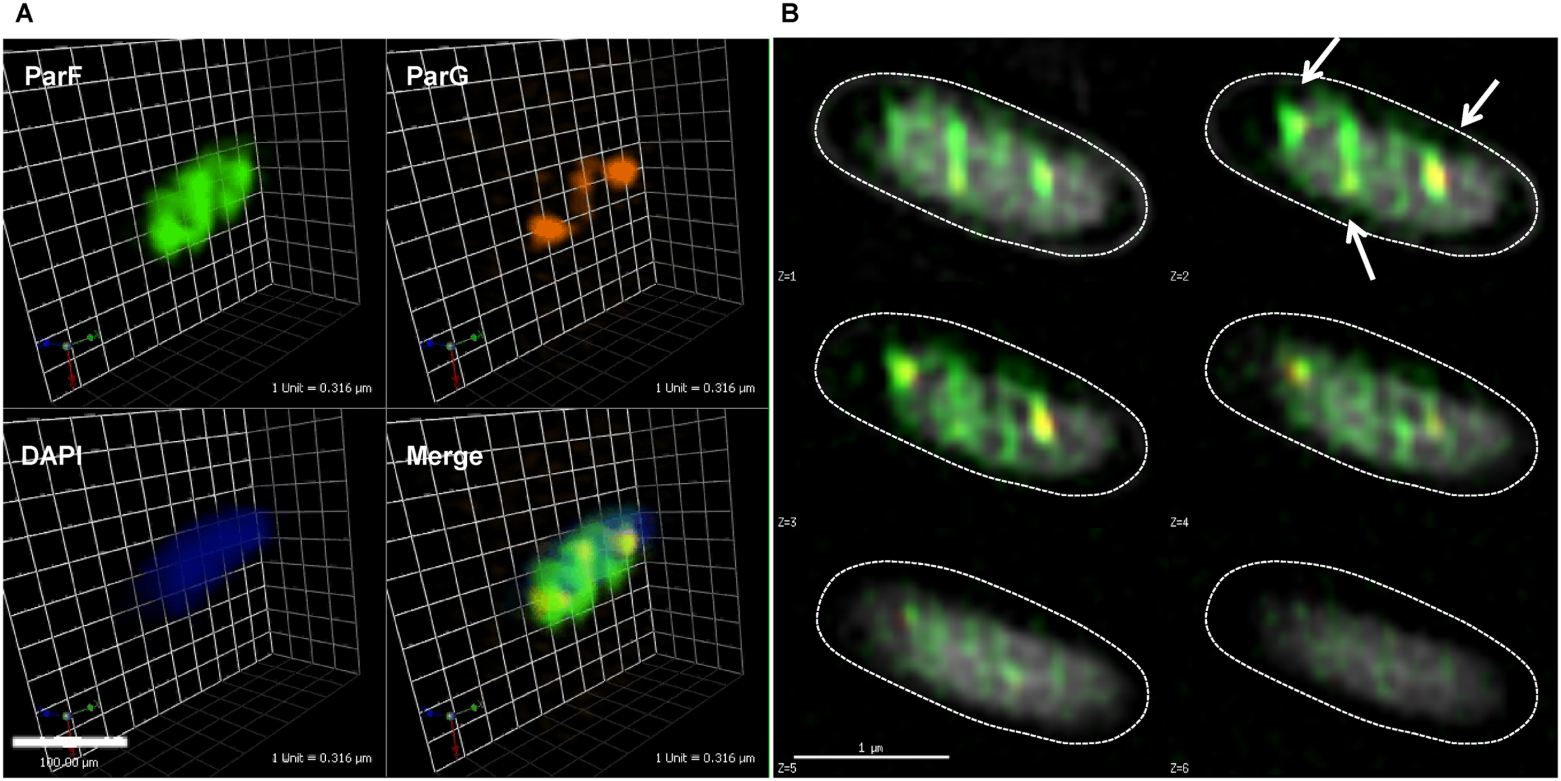
The ParF meshwork extends throughout the nucleoid interior. (**A**) Three-dimensional rendering of 3D-SIM images of a live *Escherichia coli* cell harboring the plasmid containing the *parFG-mCherry-parH* module and the plasmid expressing *parF-emerald*. Individual channels and overlay images are shown. (**B**) 3D-SIM Z-stacks of the same cell shown in panel A, the arrows indicate the ParG foci. Scale bar = 1 μm in both panels A and B.

**Figure 8.**
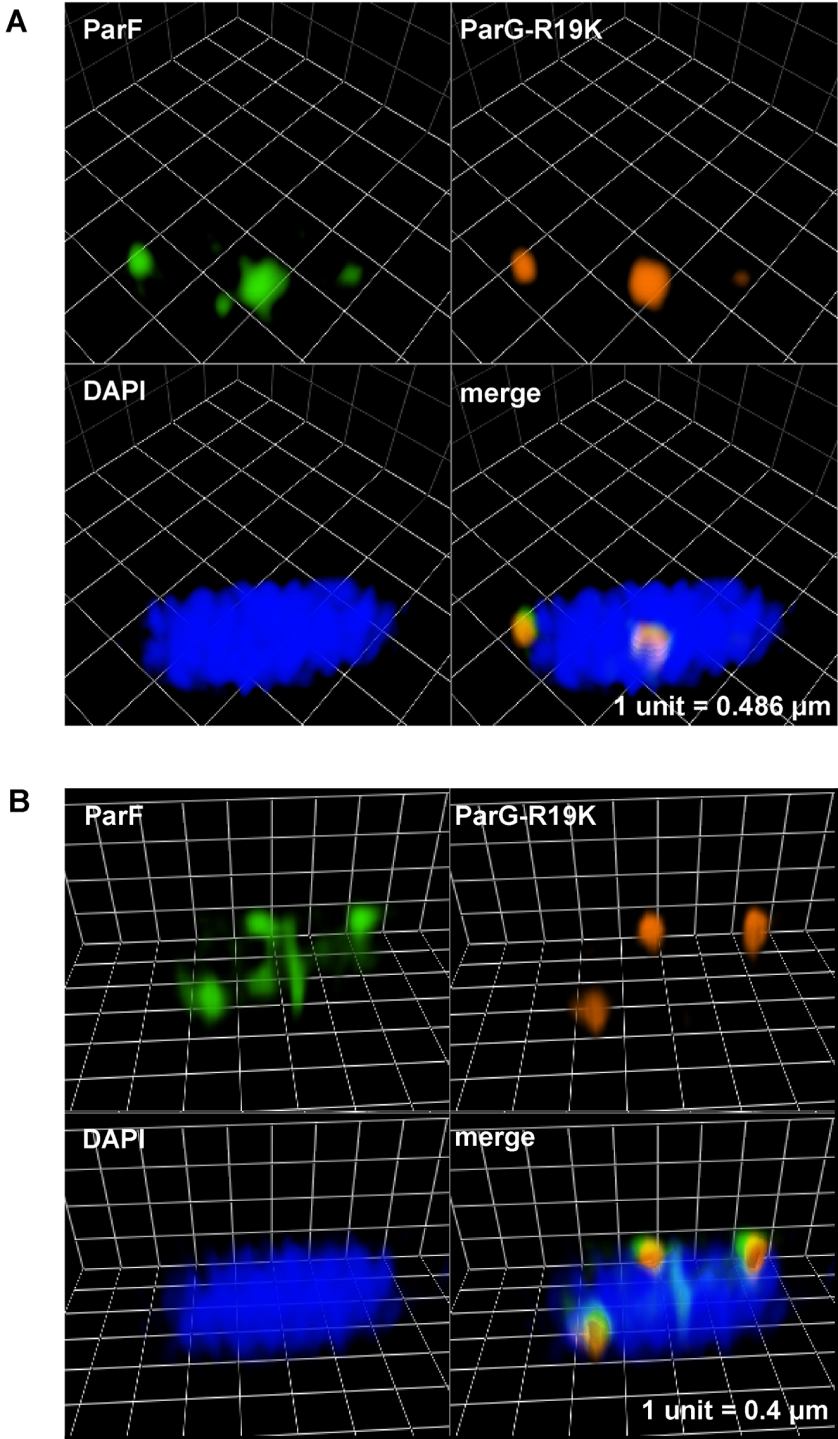
ParF coalesces into foci and does not form a meshwork in the absence of the ATP hydrolysis stimulatory activity of ParG. (**A** and **B**) Three-dimensional rendering of 3D-SIM images of *Escherichia coli* cells that carry a plasmid harboring the *parG-R19K* mutant allele in the partition module and a plasmid expressing *parF-emerald*. Individual images for green, red and blue channel are shown as well as the merge image. The nucleoid was stained with DAPI.

More detailed investigation of the ParF structure on the nucleoid was hindered by the spatial resolution limit of conventional microscopy. To achieve higher resolution of the ParF structure, we imaged cells by 3D structured illumination microscopy (3D-SIM) using an OMX microscope. Images acquired from multiple orientations are subjected to an iterative reconstruction algorithm that achieves a sub-diffraction image with a ∼2-fold increase in both *xy*- (∼100 nm) and *z*-axis (∼300 nm) resolution (41). Localization of ParF-Emerald observed by 3D-SIM was the same as that seen using conventional microscopy. In the presence of the plasmid carrying the segregation locus, the ParF signal appeared asymmetrically associated with or in transit toward one nucleoid pole (Figure 6A and B, Supplementary Figure S5 and Supplementary Movie S6). In the absence of the partition locus, ParF uniformly coated the nucleoid (Supplementary Figure S5C). However, super resolution 3D-SIM revealed that the ParF comet protrudes into the nucleoid: examination of cell cross sections showed that ParF permeates the interior of the nucleoid extending through the chromosome in three dimensions (Figure 6C). To further confirm the 3D pattern of ParF, Z-stacks were taken and clearly showed that the protein assembles into an organized, meshwork-like structure visible in the different planes of the nucleoid (Figure 7A and B). Although the ParF meshwork is visibly associated with the nucleoid, its pattern does not entirely overlap with that of the chromosome, as projections often shoot out of the nucleoid perimeter (Figure 6A and B) or fill up small pockets unoccupied by DNA (Supplementary Figure S5B). ParG foci were generally trapped within the ParF meshwork (Figure 6B), or were sometimes observed in an area largely devoid of ParF (Figure 6A, leftmost focus). Supplementary Figure S5A provides an example of a cell in which ParG foci colocalized with small patches of ParF belonging to the tail of the meshwork clustered at one end of the nucleoid. Overall, the super resolution microscopy data show that ParF assembles into a structure branching through the nucleoid volume, resulting in a 3D net through which ParG–plasmid complexes are captured and translocated. Remarkably, no ParF meshwork was observed in cells containing the ATPase stimulation-defective ParG-R19K mutant: in contrast, ParF accumulated on ParG foci (Figure 8A and B). When the non-fused green fluorescent protein's signal was imaged by 3D-SIM, it lacked specific localization and was spread throughout the cell (Supplementary Figure S6B and C). The different patterns observed for the same ParF-Emerald protein in the wild-type and ParG-R19K mutant context indicate that the ParF meshwork is a physiologically relevant, genuine structure of ParF that is crucial for plasmid segregation. The data also show that the meshwork is not the result of ParF fusion to the Emerald protein, because if it were, then the ParF-Emerald pattern would be identical in both backgrounds. ParF-G179A assembled into a structure similar to that of wild-type ParF (Supplementary Figure S6A).

### ParF harbors a cluster of positively charged residues and associates with non-specific DNA *in vitro*

**Figure 9.**
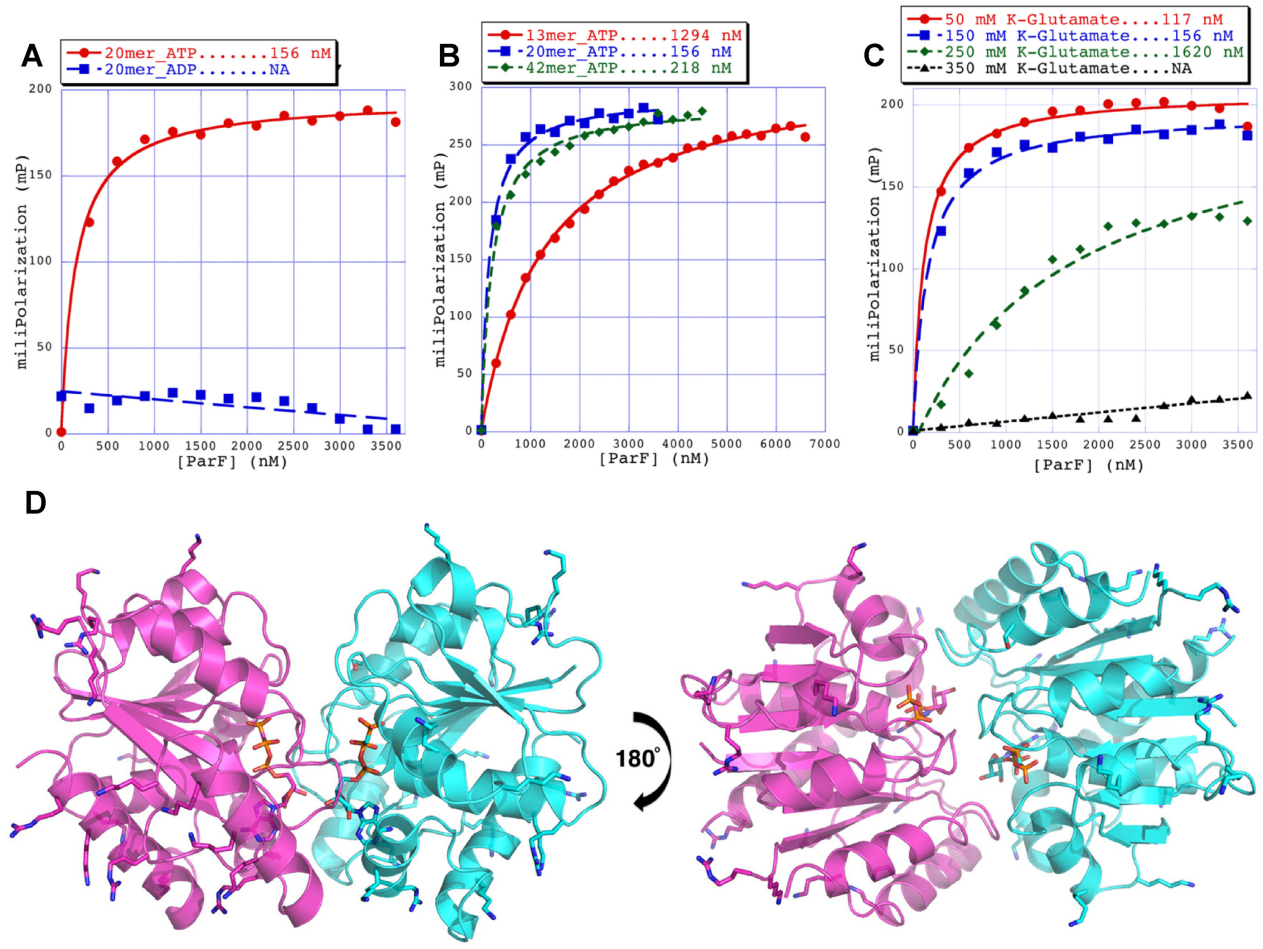
ParF associates with non-specific DNA. Fluorescence polarization studies performed with ParF and fluoresceinated oligonucleotides harboring a random sequence. Analysis of DNA binding in the presence of different nucleotides (**A**), different sizes of oligonucleotides (**B**) and different salt concentrations (**C**). (**D**) Structures of the the nucleotide sandwich ParF dimer showing the plethora of basic residues that cover the dimer surface that could be used to interact non-specifically with DNA. Basic side chains and the AMP-PNP molecules are shown as sticks.

Microscopy indicated that ParF localizes on the nucleoid. To investigate whether the protein binds DNA, fluorescence polarization experiments were carried out using ParF and fluoresceinated double-stranded oligonucleotides harboring a random sequence. First, ParF bound a 20-mer oligonucleotide with high affinity in the presence of ATP with a *K*_d_ of 156 nM, but did not bind in the presence of ADP (Figure 9A). These findings establish that ParF association to DNA is reliant on ATP binding. Second, we examined the length necessary to allow binding by using 13-, 20- and 42-mer oligonucleotides in the presence of ATP. ParF loosely associated with the 13-bp site, but avidly bound the 20- and 42-bp oligonucleotides (Figure 9B). Thus, ParF DNA binding is dependent on the length of the fragment. Third, the salt dependence of the ParF–DNA interaction was explored. Binding of ParF–ATP to the 20-mer DNA site was measured in the presence of increasing concentrations (50–350 mM) of potassium glutamate. ParF bound DNA with a *K*_d_ in the nanomolar range up to 150 mM potassium glutamate (Figure 9C). However, higher ionic strength inhibited binding, which is consistent with non-specific DNA binding by ParF.

Inspection of the ParF structure revealed that multiple basic residues coat the surface of the protein (Figure 9D) and are thus optimally positioned to associate with the DNA and allow ParF to become enmeshed within the nucleoid.

## DISCUSSION

ParA proteins are responsible for plasmid translocation and maintaining plasmid position during cell growth (2,42). This is a problem of motion, spatial arrangement and tempo. Here we have established that no spatial positioning of plasmids carrying the *parFGH* module is attained in the absence of a functional ParF (Figure 1). Cells harboring defective ParF-K15Q and ParF-G11V proteins show a single, randomly located ParG–plasmid focus, suggesting that post-replication separation of sister plasmids is not achieved. Thus wild-type ParF initiates the segregation process by splitting paired plasmids and then defining their subcellular coordinates. Like other ParA family members, ParF binds DNA non-specifically (Figure 9) and localizes to the nucleoid (Figures 2 and 6 and Supplementary Figure S3). Due to a cooperative ATP-induced assembly into higher order structures, ParF grows into a 3D meshwork through the nucleoid, as established by 3D-SIM experiments (Figures 6 and 7 and Supplementary Figure S5). This allows ParF to survey the nucleoid territory, confining plasmid foci to that region only and demarcating the area of the cell proficient for plasmid segregation. The transient, dynamic nature of the polymers ensures continuous remodeling of the ParF structure, so that more distant positions can be reached, upon nucleoid growth and elongation. Multiple cross-nucleoid oscillations may periodically adjust the 3D coordinates of plasmids within the elongating nucleoid. Thus ParF functions as a 3D molecular scanner within the nucleoid perimeter.

ParA ATPase activity is enhanced by its partner protein (4–10,13,28). An arginine finger-like motif in the N-terminus of ParG stimulates ParF ATP hydrolysis and is required for accurate plasmid segregation (35). Here, we have established that no relocation of the ParF meshwork occurs in the absence of a functional ParG and *parH* site (Supplementary Figure S4A and B). When ParG is provided *in trans*, ParF shuttling across the nucleoid resumes (Supplementary Figure S4B and Supplementary Movie S4). Thus, ParG triggers the sequence of events leading to plasmid segregation. The most likely mechanism whereby ParG may ignite ParF relocation is through stimulation of ATP hydrolysis that would remove ParF from the DNA leading to dynamic remodeling of the ParF bundles. To investigate this hypothesis, cells that harbored a plasmid encoding the stimulation-impaired ParG-R19K mutant were imaged. Although ParF is wild-type, neither meshwork structure nor oscillation over the nucleoid were detected (Figures 4B and 8). This indicates that the dynamic behavior of ParF is dependent on stimulation of its ATPase activity by ParG. Interestingly, ParF forms discrete foci that overlap those of ParG-R19K, showing that ParF interacts with and accumulates on ParG–R19K–plasmid complexes (Figures 4B and 8). However, due to lack of ATP hydrolysis stimulation, no ParF meshwork assembly-disassembly cycles occur resulting in lack of transport and positioning of plasmid complexes. The ∼30% of cells harboring a single plasmid focus at the mid-cell position are likely to be cells in which the ParG–R19K–plasmid cluster is located between separated nucleoids (Figure 1C). Significantly, the observation that wild-type ParF displays different patterns in the ParG and mutant ParG-R19K backgrounds demonstrates that the ParF meshwork is a physiologically relevant structure and not a counterfeit resulting from the fusion to the Emerald fluorescent protein.

A different insight into the importance of temporal regulation of segregation events was provided by the ParF-G179A mutant that shows hyperactive ATP hydrolysis. Counter-intuitively, ParF-G179A and ParG-plasmid localization patterns are indistinguishable from those observed in the wild-type background and ParF-G179A is capable of cooperative assembly into a meshwork apparently equivalent to that formed by wild-type ParF (Supplementary Figure S6). However, what is aberrant in this context is the tempo: ParF-G179A oscillation dynamics are faster as a result of its hyperactive ATP hydrolysis (Figure 5). ParF-G179A interacts with ParG, but no longer responds to its stimulatory effect (40). The unregulated ATPase kinetics change the tempo of oscillation and abrogate plasmid partition. Although plasmid foci are split and apparently positioned, they might not be pinned down and held at those locations at the time of cell division due to the very dynamic nature of ParF-G179A and the altered interaction with ParG. The overall conclusion is that ParG acts as a metronome that dictates the tempo of segregation events, and that nucleoid localization and oscillation *per se* do not ensure plasmid segregation in the absence of finely tuned ATPase kinetics.

The current picture of plasmid segregation mediated by ParA proteins is quite fragmented and the mechanism is still elusive. Different models have been proposed whose common denominator is the role of the nucleoid as a platform for segrosome attachment. Based on findings for pB171 partition, one model invokes the formation of a nucleoid-associated ParA filament whose growing tip stochastically captures a ParB–plasmid complex. Upon ATP hydrolysis stimulated by ParB, the ParA filament disassembles pulling the plasmid toward one pole of the nucleoid (20). The ParA structure is referred to as a filament, based on *in vitro* evidence demonstrating ParA protein polymerization and on *in vivo* microscopy experiments showing an elongated ParA shape. Based on *in vitro* studies, another model proposes a diffusion-ratchet mechanism in which ParA recruits the ParB–plasmid cargo to the nucleoid and a ParB-induced ParA gradient guides plasmid movement (23,27,29,30,47). This model postulates movement of ParA and ParB–plasmid molecules on the surface of the nucleoid and numerous studies performed with components of the P1 and F plasmid systems and a flat surface DNA carpet have generated the tenets of this predicted mechanism in a cell-free setup. More recently, a modified version, designated as DNA-relay model, was proposed (28). In analogy to the Brownian ratchet put forward for plasmid systems, the mechanism envisaged by these authors involves a gradient of chromosomally-encoded ParA in *C. crescentus* cells that is responsible for the translocation of ParB bound to *parS* across the cell. However, based on simulations of partition complex diffusion and mathematical modeling, the DNA-relay model predicts that diffusion alone is not sufficient to mediate movement of the partition complex and that instead the elastic properties of the chromosome propel the complex across the ParA gradient (28).

Based on our experimental observations, we propose a new model to rationalize the mechanism of plasmid segregation by ParA proteins. Using conventional fluorescence microscopy, a filament-like structure for ParF is not apparent in live cells, even after deconvolution. Instead a diffuse structure is observed (Figures 2 and 3; Supplementary Figure S3B). A cloud-like signal has been observed for a number of plasmid and chromosome encoded ParAs. Super resolution 3D-SIM revealed that the ParF structure is a 3D meshwork branching out into the nucleoid (Figures 6 and 7; Supplementary Figure S5 and Supplementary Movie S6). Biochemical *in vitro* investigations and structural data have provided evidence of the highly cooperative self-assembly nature of ParF (8,34,35,40). In the super resolution images the ParF signal coalesces into interconnected patches and bundles that permeate the interior of the nucleoid, but also fills in nucleoid gaps and protrudes from its surface. Interestingly, a study investigating ParA of plasmid pB171 suggested that the protein might form structures within the nucleoid rather than homogeneously covering its surface (43). In addition, while this work was being prepared for submission, a paper that provided evidence for plasmid segregation through the nucleoid volume was published (44). Recent studies have reported that the *E. coli* chromosome is a low-density ellipsoid, whose radial confinement imparts on it a helical shape (45,46). Super resolution images of the ParF meshwork seem to suggest that the structure has a helical pitch that might be a reflection of the underlying helical chromosome shape. However, this feature needs to be further investigated.

**Figure 10.**
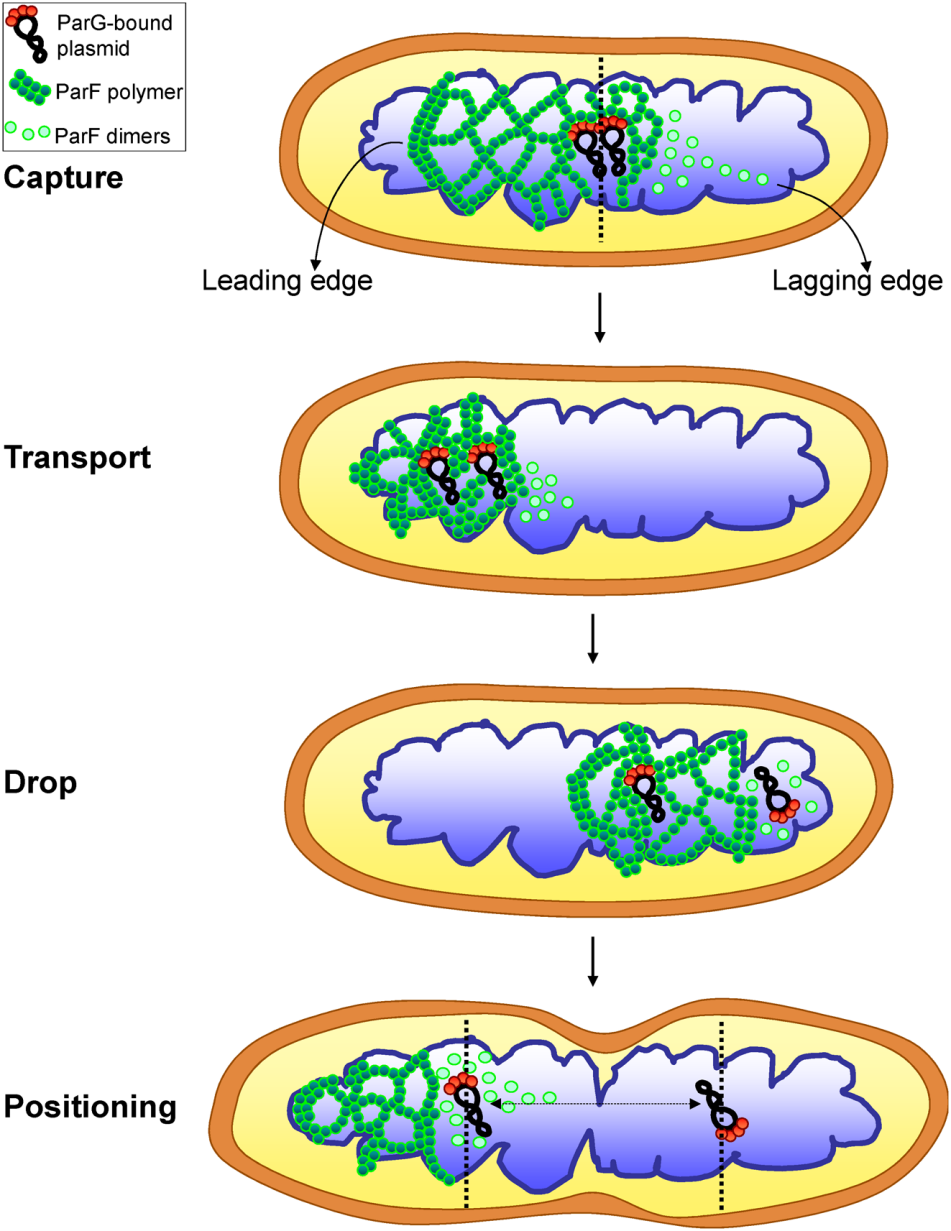
A model for ParF meshwork-mediated plasmid segregation. A pair of newly replicated plasmids carrying ParG dimers bound to the partition sites is engulfed into the ParF meshwork, anchored to it via the interaction of ParG with ParF and transported toward one pole of the nucleoid. The ParF meshwork exhibits a leading edge that is more compact corresponding to the advancing front and a looser weave tail that consists of less densely packed oligomers. During the journey across the nucleoid, a network of intersecting polymer bundles grows between the plasmids until one plasmid becomes detached from the lagging edge due to ParG-stimulated ATP hydrolysis and dropped at one pole of the nucleoid. The sister plasmid remains anchored to the ParF meshwork and is transported to the opposite end of the nucleoid, where it is ultimately released. Subsequent rounds of the ParF meshwork oscillation reset the position of the two plasmids within the elongating nucleoid so that they are driven to the center of the replicated chromosomes.

What are the implications of a ParF meshwork for plasmid segregation? A 3D meshwork acts as a ‘Venus flytrap’ which ensures that ParG–plasmid complexes are readily captured by ParF and is a more effective strategy than random collisions of single ParA molecules or a linear filament with plasmid complexes. Given the crowded milieu of the cell and the low copy of plasmids, it is crucial that the system is efficient. Based on biochemical and structural data (8,34), we interpret the ParF meshwork as a network of intersecting polymer bundles that result from ParF self-association and ramify in multiple directions, protruding into the nucleoid. A meshwork of polymers imparts higher flexibility as it offers multiple attachment points for cargos not only in the XY- but also in the Z-dimension of the nucleoid. Moreover, oligomers at different points of the meshwork can undergo localized disassembly induced by ParG resulting in plasmid release. Additionally, a 3D structure guarantees surveying the entire nucleoid volume, not simply the nucleoid surface as proposed by other models (23,30,47). Pairing of newly replicated plasmids at midcell is thought to be an initial event during partition (48). The plasmid pair may then be engulfed into the ParF meshwork via interaction with ParG dimers bound to the partition site and transported to one pole of the nucleoid (Figure 10). The ParF meshwork presents a leading edge that corresponds to the compact, advancing front and a looser-weave, lagging tail that consists of less densely arranged oligomers. As plasmids are transported, a network of branched ParF polymers grows between them until one plasmid is dropped due to localized ATP hydrolysis mediated by ParG that causes filament disassembly at the lagging edge of the meshwork. The sister plasmid remains tethered to the compact edge of the ParF meshwork and is shifted to the opposite end of the nucleoid, where eventually it is released again due to ParG-mediated remodeling of the edge of the meshwork. Subsequent rounds of oscillation of the ParF mesh ensure small-scale adjustments of plasmid position to the middle of the future nucleoids prior to cell division. The repeated cycles of capture and release are important to reset the position of the plasmids over the elongating nucleoid. When the oscillation speed of ParF becomes altered due to aberrant ATP kinetics, the separated plasmids might not maintain their final position during the subsequent process of cell division. This model describes a mechanism that is not antithetical to earlier hypotheses, but that amalgamates the concepts of ParA higher-order structures and gradient invoked by previous models. In all cases, the ParA protein defines the territory for plasmid segregation, while ParB/ParG functions as timekeeper setting the timing for relocation of ParA molecules across the nucleoid. As numerous ParA proteins involved in DNA and protein trafficking (49,50) have been visualized as diffuse, asymmetric clouds by conventional microscopy, we speculate that a 3D meshwork might be a widespread mechanism utilized by Walker type ATPases to distribute and position sizeable cargos in prokaryotes.

## Supplementary Material

Supplementary Data
