# Supplementary material for: A three-dimensional ParF meshwork assembles through the nucleoid to mediate plasmid segregation
Source: Nucleic Acids Res. 2016 Dec 29;45(6):3158–71. doi: 10.1093/nar/gkw1302 (PMC5389482; doi:10.1093/nar/gkw1302)
Supplement: Supplementary Data [file gkw1302_Supp.zip › nar-02253-m-2016-File012.docx]

**SUPPLEMENTARY DATA**

**A three-dimensional ParF meshwork assembles through the nucleoid to mediate plasmid segregation**

Brett N. McLeod, Gina E. Allison-Gamble, Madhuri T. Barge, Nam K. Tonthat, Maria A. Schumacher, Finbarr Hayes and Daniela Barillà

**Supplementary Figures**

**Figure S1.** Western blots on *E. coli* cells that harbour the two-plasmid system used for microscopy experiments. Cultures were grown under the same conditions used for microscopy as described in Materials and Methods. Aliquots of the extracts and of purified ParF **(A)** and ParG **(B)** were subjected to gel electrophoresis on 12% SDS-polyacrylamide gels that were then immunoblotted with affinity-purified anti-ParF and anti-ParG antibodies.


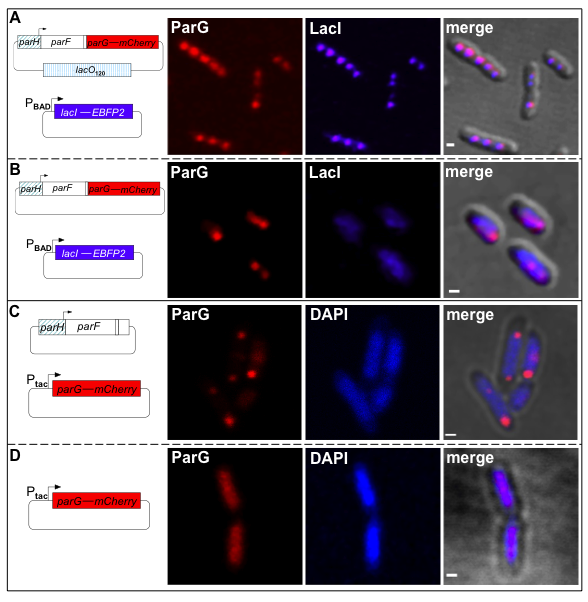


**Figure S2.** ParG colocalizes with plasmids harboring the *parFGH* module. **(A)** Fluorescence microscopy images of *E. coli* cells cotransformed with a segregation probe containing the *parFG-mCherry-parH* module and a *lacO*_120_ array and a plasmid expressing *lacI-ebfp2* from an arabinose inducible promoter. ParG and LacI foci are red and blue, respectively. **(B)** *E. coli* cells cotransformed with the plasmid harboring the *parFG-mCherry-parH* module (and no *lacO* array) and the plasmid expressing *lacI-ebfp2* from an arabinose inducible promoter. **(C)** *E.coli* cells containing a plasmid harboring a *parF*Δ*parG-parH* module and a plasmid expressing *parG-mCherry* from the P_tac_ promoter in the absence of induction. **(D)** *E. coli* cells harboring a plasmid expressing *parG-mCherry* from the P_tac_ promoter in the absence of induction. All cultures were grown in M9 glucose in the presence of 0.02% L-arabinose. In both C and D, the nucleoid is stained with 4', 6-diamidino-2-phenylindole (DAPI).

Scale bar = 0.5 μm in all panels. The rightmost panels show the merge of bright field and fluorescent signals.

**Figure S3.** Subcellular localizations of ParG and wild type or mutant ParF relative to the nucleoid. **(A)** *E. coli* cells were transformed with a plasmid expressing *parF-emerald* or the indicated mutant allele from the P_BAD_ promoter and observed by microscopy. ParF-Emerald localization (top row) and nucleoid (DAPI stain, middle row) were merged with a bright field channel image to show localization of ParF-Emerald proteins relative to nucleoid and cell boundaries (bottom row). Scale bar = 0.5 μm.

**(B** and **C)** *E. coli* cells transformed with plasmids, where the indicated *parF* allele was expressed from the *parFG-mCherry-parH* module, and with a plasmid expressing the same wild type or mutant *parF-emerald* allele. Cells were grown in M9 glucose supplemented with 0.02% L-arabinose. Scale bars = 0.5 μm.

**Figure S4.** ParF does not oscillate in the absence of ParG and the partition site *parH*. **(A)** Fluorescence microscopy time-lapse images of *E. coli* cells expressing *parF-emerald* from the P_BAD_ promoter in the absence of TP228 partition module, scale bar = 1 μm. **(B)** Microscopy time-lapse images of *E. coli* cells harboring a plasmid expressing *parF-egfp* from the P_BAD_ promoter and (*top row*) a plasmid containing a *parF*Δ*G-H* partition module, (*middle row*) a plasmid containing a *parF*Δ*G-H* partition module + a plasmid expressing *parG-mCherry* from the P_tac_ promoter, (*bottom row*) a plasmid expressing *parG-mCherry* from the P_tac_ promoter. Cell boundaries (dashed lines) were overlaid from bright field images. Scale bar = 0.5 μm.


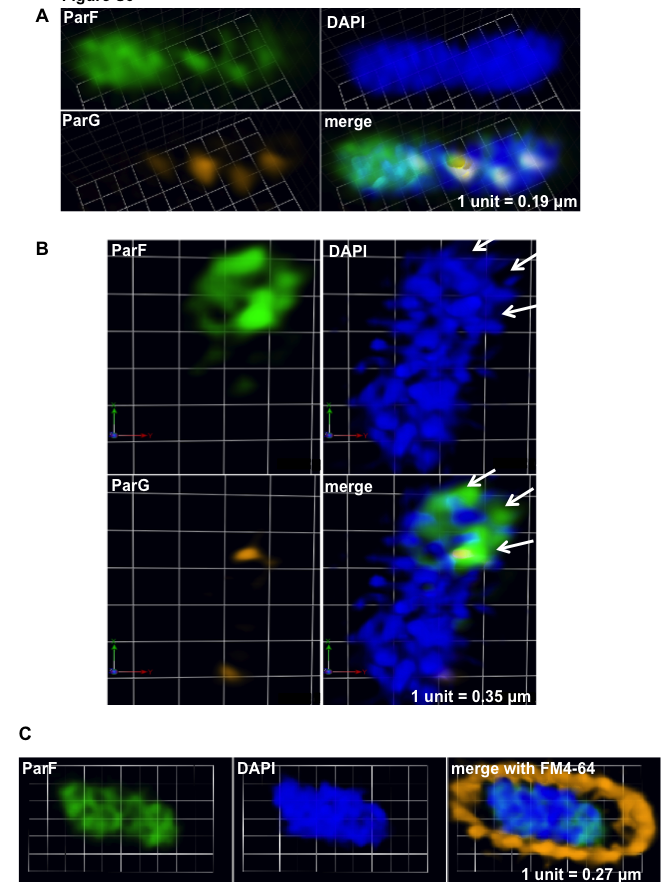


**Figure S5.** A ParF meshwork assembles through the nucleoid. Three-dimensional rendering of 3D-SIM images of *E. coli* cells acquired with the OMX microscope. Individual images for green, blue, red channel are shown as well as the merge image. The nucleoid was stained with DAPI. **(A** and **B)** Cells harboring the plasmid carrying the *parFG-mCherry-parH* module and a plasmid expressing *parF-emerald* from the P_BAD_ promoter. The arrows point to small pockets in the nucleoid weave that are filled in by ParF. **(C)** Cell expressing *parF-emerald* from the pBAD construct in the absence of the TP228 segregation locus. The merge image shows ParF, the nucleoid and FM4-64-stained membrane.

**Figure S6.** ParF-G179A assembles into a three-dimensional structure analogous to that formed by wild type ParF. **(A)** Three-dimensional rendering of 3D-SIM images of *E. coli* cells acquired with the OMX microscope. Cells carry a plasmid harboring the *parF-G179A* mutant allele in the partition module and a plasmid expressing *parF-G179A-emerald*. Individual images for green, red and blue channel are shown as well as the merge image. The nucleoid was stained with DAPI. **(B** and **C)** Three-dimensional rendering of 3D-SIM images of *E. coli* cells carrying a plasmid expressing the gene for GFP alone.

**Supplementary movies legends**

**Movies S1 and S2.** The ParG focus is excluded from the nucleoid in the absence of ParF. Live *E. coli* cells harbouring the plasmid that carries the *parΔF-parG-mCherry-parH* module and the pBAD30 plasmid. The nucleoid was stained with DAPI. The overlay of bright field, red and blue channel is shown. Scale bar = 1.2 μm.

**Movie S3.** Dynamic relocation of ParF in live cells. Live *E. coli* cells harboring the plasmid that carries the *parFG-mCherry-parH* module and the plasmid encoding ParF-Emerald were imaged for 20 minutes. The overlay of bright field and green channel is shown. Scale bar = 1 μm.

**Movie S4.** Reconstitution experiment in which providing ParG *in trans* restores ParF dynamic relocation. Live *E. coli* cells carrying a plasmid containing a *parF*Δ*G-parH* partition module, a plasmid expressing *parF-emerald* from the P_BAD_ promoter and a plasmid expressing *parG-mCherry* from the P_tac_ promoter were imaged for 11 minutes. The overlay of bright field, green and red channels is shown. Scale bar = 1 μm.

**Movie S5.** The hyperactive ATP hydrolysis of ParF-G179A results in increased frequency of oscillation. Live *E. coli* cells harboring a plasmid that carries the TP228 partition locus encoding the ParF-G179A mutant and a plasmid expressing *parF-G179A-emerald* from the P_BAD_ promoter were filmed for 20 minutes. The overlay of bright field and green channel is shown. Scale bar = 1 μm.

**Movie S6.** A ParF meshwork encases the nucleoid. Three-dimensional rendering of a 3D-SIM Z-stack showing ParF, ParG and nucleoid in an *E. coli* cell rotated on its long axis. *E. coli* transformed with a plasmid carrying *parFG-mCherry-parH* and a plasmid expressing *parF-emerald* from the P_BAD_ promoter was imaged by 3D-SIM using an OMX microscope. Volocity imaging suite (Perkin Elmer) was used to construct the rotation movie.
